# Comparison of Effects of Labetalol and Nitroglycerine on Intraoperative Blood Loss and Surgical Field Quality in Rhinoplasty Surgery

**Published:** 2015-01

**Authors:** Mohamad Reza Hadavi, Yadollah Zarei, Shojaolhagh Tarogh

**Affiliations:** Department of Anesthesiology and Intensive Care, Shiraz University of Medical Sciences, Shiraz, Iran

**Keywords:** Rhinoplasty, Labetalol, Nitroglycerin, Intraoperative bleeding

## Abstract

**BACKGROUND:**

Rhinoplasty is one of the most common surgeries of the plastic surgery and as well as ear, throat and nose. Intra-operative bleeding during surgery is one of the most important factors that may impair the surgeon’s job. Providing a clean blood-free surgical filed makes the operation faster, easier and with a better quality. One way to achieve this goal is to induce hypotension. This study aimed to compare the impacts and outcomes of administration of labetalol or nitroglycerin for this purpose.

**METHODS:**

In this randomized clinical trial, 60 ASA I and ASA II patients who were referred for rhinoplasty were enrolled. Patients were randomly assigned to two groups. Labetalol was given to the first and nitroglycerin to the second group of patients. Blood pressure and the amount of intra-operative bleeding during surgery and surgeon satisfaction were measured.

**RESULTS:**

The average age of patients was 25.9±7.52 years. The average amount of bleeding among all patients was 117.87±324.86 ml, and the average quality of the surgical site was 1.65±4.48, considering all patients. The average quality and average surgical site bleeding between the two groups was not significant.

**CONCLUSION:**

There was a little difference between labetalol and nitroglycerine on the effect of intraoperative blood loss and surgical field quality in rhinoplasty surgery.

## INTRODUCTION

Induction of hypnosis, and preparing a pain free state for the patient with no body movements are regarded as the most important benefits of applying anesthesia. However Anesthesiologist can also improve the quality of operation by preparing a clear view for the surgeon at the operation zone.^1^ One of the major obstacles in major surgeries that can highly affect the quality of operation and its demanded time, is the non-stop bleedings at the target site of surgery. This issue can seriously limit the surgeon’s maneuver over the target tissue and can cause non-desired invasions to neighboring organs.^2^ Rhinoplasty is one of the most common cosmetic surgeries among the surgeries of ear, throat and nose. Although this kind of surgery is not associated with major bleeding, but clearing the small operation site demands multiple suctions that can increase the bleeding and damage surrounding tissues.^2^

In this regard, several approaches have been used to reduce the bleeding amount although none of them has shown priority over others. These methods include simple ones like positioning the head higher than the heart surface^1^ or administration of vasoconstrictive agents,^3^ induced hypotension^4^ and intravenous anesthesia administration.^5^ Through the operation, the anesthesiologist should prepare a stable condition for surgery, take care of air ways and make sure of proper ventilation of lungs besides the patient’s anesthetized condition.^6^ All of the above criteria can be easily monitored through the general anesthesia process. 

However administration of the related anesthetic agents can increase the bleeding amount compared to using local anesthesia.^7^^,^^8^ This can limit the surgeon’s view of the target tissue which can increase the risk of damage to eye vessels or post-operation inter-cranial complications.^7^^,^^9^^,^^10^ The high perfusion rate of head tissue makes it susceptible to major bleedings even due to minor injuries and this is especially important in several anatomical zones like anterior ethmoid, sphenopalatine artery branches and the posterior zone of inferior concha which account for most of bleeding problems during rhinoplasty.^7^

The average arterial blood pressure is one of the most important factors in determining the bleeding amount during surgery which is under the effect of heart functionality and output or the general resistance of vessels against blood stream.^11^^,^^12^ Heart output can change upon autonomous nervous system stimulation, the speed of impulse transduction through heart tissue and the myocardium contraction power. If the parasympathetic system effect is dominant, the heart rate slows down, the speed of impulse transduction through AV node decreases and the contractive power of myocardium weakens.^13^ On the other hand parasympathetic stimulation can cause vasodilation, decreased blood pressure and reduced heart output.^14^

One of the successful approaches in reducing the bleeding amount is controlled reduction of systolic blood pressure to 50 mmHg which has no major side effects in healthy cases. Several drugs have been administered in this regard among which sodium nitroprusside, nicardipine, nitroglycerin and beta-blockers are commonly used.^14^ Some of inhaling anesthetics like halothane and enflurane can also have vasodilating effects.^14^

Propofol is one of the IV administered hypnotic agents during rhinoplasty which can prepare a better situation compared to inhaling anesthetic use. This agent cause reduced brain and arterial circulation.^8^ In this regard, the blood circulation of ethmoid and sphenoid is restricted and a suitable environment for surgery is resulted. Opioids can also cause decreased blood pressure and reduced hemodynamic responses to surgery triggered stresses which increase the bleeding problem.^15^ Sufentanil and remifentanil prepare a more suitable hemodynamic environment for the surgery compared to other commonly used opioids.^16^ Administration of these drugs with high doses of benzodiazepines decreases the systemic blood pressure and heart output. These agents also facilitate the action of other drugs with blood pressure lowering effects.^16^

In spite of the mentioned methods for bleeding control, the most common approaches for reducing the bleeding problem during rhinoplasty were pre-operational administration of nasal anti-congestions with oxymetazoline, cocaine and local adrenaline, through nasal pack or bipolar cautery;^1^^,^^3^ infiltration into nasal lateral wall with 0.008 adrenaline containing lidocaine;^17^ and fibrin glue and the products that contain a mixture of biological coagulants like thrombin, fibrinogene and cryoprecipitate.^18^ These methods have considerable limitations like transfer of pathogens through biological products or high production cost^18^^,^^19^ while thrombin or collagen use can promote scar tissue formation.^20^^,^^21^ Use of nasal packs may also cause increased bleeding after the pack removal.^22^

Labetalol is an antagonist of adrenergic receptors (a1, b1, b2) and is used as an hypotension inducer. This drug targets the b receptors 5 to 10 times more specific than a receptors so that minor tachycardia happens upon its administration.^23^ Nitroglycerin can also cause hypotension through its vasodilation effect on veins. However this drug causes increased heart rate as a non-desirable side effect.^24^ Although both drugs have been used in different surgeries to control the bleeding issue and with acceptable results,^23^^,^^25^^-^^28^ there are no comparative data in the case of rhinoplasty. 

Regarding their distinctive mechanism, these two drugs may have different effects on the bleeding amount at the field of surgery. In the current study, we tried to see if such distinction can be seen in the case of rhinoplasty or not. Regarding the fact that during the surgery a low heart rate with no tachycardia is desired, our results can be important for the future drug choices to limit the bleeding problem during rhinoplasty. This study aimed to compare the impacts and outcomes of administration of labetalol or nitroglycerin for this purpose. 

## MATERIALS AND METHODS

Sixty patients who were undertaken for rhinoplasty and had no previous history of bleeding problems, uncontrolled blood pressure issues, brain vascular diseases, coronary diseases, cardiac dysrhythmia, liver and kidney problems or metabolic disorders were enrolled. None of the cases were consuming the drugs that affect homeostasis and none of the patients were pregnant. All of the enrolled cases were ASA I or ASA II patients. Our target population was randomly divided into two groups (using www.randomizor.org website) with equal number of patients and each group received labetalol (L group) or nitroglycerin (N group) to control the bleeding issue. All of the enrolled patients were between 17-45 years old and all of them had normal platelet and coagulation test results.

All patients received 0.03 mg/kg of midazolam and 2 mg/kg of fentanyl as the pre-medication. Then the anesthesia was induced with 2 mg/kg of propofol, 0.15 mg/kg of atracurium and 0.1 mg/kg of morphine. As the patient entered the anesthetic state, the infusion of propofol was started with the speed of 100-200 μg/kg/min to stable the anesthetic state.

Prior to the surgery incisions, 0.4-3 mg/kg/h of labetalol (L group) and 0.5-5 μg/kg/min of nitroglycerin (N group) was administered to keep the average arterial blood pressure around 55-65 mmHg. Blood pressure was measured with oscillatory method using the arm cuff every 5 minutes and heart beat rates were measured based on the pulse oximetry method. In order to measure the bleeding amount at the field of surgery, a suction device with small tank was used. We also recorded the amount of absorbed blood to the packs that were used to dry the field of surgery. The patient’s information, time of surgery, bleeding amount, average arterial pressure, heart beat and the amount of administered hypotension inductive drugs were registered by the anesthesiologist who knew nothing about the aim of our study. Evaluation of surgery field quality was fulfilled by a surgeon who was ignorant about type of administered hypotensive drug. This was achieved by filling the Boezaart table^29^ which is a scoring table to measure the quality of surgery. In the case of patient’s abnormal reaction to the administered drugs, which lead to the protocol change, that patient was excluded from our study.

Data are reported with descriptive statistic terms like mean±SD and frequency (percentage). Independent T-test or Mean-Whitney-u test and Chi-Square test were used to compare the results of each group. The differences between parameters were considered significant at P<0.05 and all analysis were done with SPSS software (Version 19, Chicago, Il, USA).

## RESULTS

The average age of patients was 25.9±7.52. This average was 26.1±7.42 in L group and 25.7±7.42 in T group. Age average had no significant difference between the two groups (P=0.93). Both groups were consisted of 7 men (23.3%) and 23 women (76.7%). The average weight of patients was 60.96±10 kg in L group and 63±11.62 kg in N group. However no significant difference was observed between the two group’s weights. (Table 1)

**Table 1:** Demographic information of labetalol (L group) and nitroglycerin (N group) groups

**Demographic information**		**N group**	**L group**	**P value**
Average age (years)		25.7±7.42	26.1±7.42	0.93
Average weight (kg)		60.96±10	63±11.61	0.84
Patients’ sex	Male	7 (23.3%)	7 (23.3%)	
	Female	23 (76.7%)	23 (76.7%)	
Patients’ weight (kg)	<50	3 (10%)	4(13.3%)	
	50-75	24 (80%)	24 (80%)	
	>75	3 (10%)	2 (6.7%)	

The bleeding amount was summarized in table 2. The average bleeding amount was 347.86±129.87 ml in L group while in N group this value was 301.76±101.57 ml. However, no significant difference was observed between the two groups (P=0.52).

**Table 2:** Bleeding amount distribution between labetalol (L group) or nitroglycerin (N group) groups

**Bleeding amount**	**L group** ** (N=30)**	**N group (N=30)**	**Total (N=60)**
<200 ml	3 (10%)	6 (20%)	9 (15%)
>200 and <350 ml	16 (53.3%)	13 (43.3%)	29 (48.3%)
<350 ml	11 (36.7%)	11 (36.7%)	22 (36.7%)

Among all patients, 19 cases gained a score between 2 and 3 while 28 subjects had a score between 4 and 5, ten cases were between 6 to 7, and for 3 individuals, the score was between 8 and 9 (Table 3).

**Table 3:** Distribution of operation site quality between labetalol (L group) or nitroglycerin (N group) groups

**Quality score**	**L group (N=30)**	**N group (N=30)**	**Total (N=60)**
2-3	8 (26.7%)	11 (36.7%)	19 (31.7%)
4-5	14 (46.7%)	14 (46.7%)	28 (46.7%)
6-7	7 (23.3%)	3 (10%)	10 (16.7%)
8-9	1 (3.3%)	2 (6.7%)	3 (5%)

**Fig. 1:**
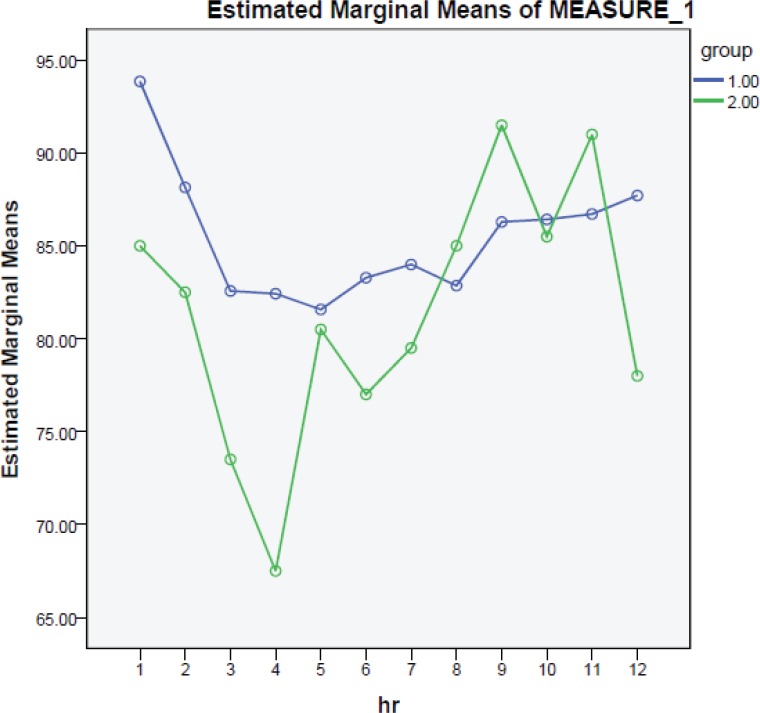
Average rate of heart beat per minute in every 15 minutes for labetalol (group 1) and nitroglycerin groups (group 2).

However the average quality of operation field showed no significant difference between the two groups with L group showing an average score of 4.66±1.70 and N group showing the 4.30±1.60 average for the quality of operation field (P=0.49). Figure 1 shows the heart beat average in each group every 15 minutes. The average of heart beats were 85.488 and 81.48 per minute in L and N groups respectively and no significant difference between the two groups was observed.

## DISCUSSION

Preparation of a suitable operation field with a clean view and appropriate homeostatic condition is one of the important goals in management of anesthetic state of patients. Induced hypotension can lower the bleeding amount and hence is one of the major approaches to achieve such conditions. In this regard, the bleeding amount will be efficiently reduced and the surgeon can have a better view of operation site. Several drugs like nitroprusside, nitroglycerin, labetalol and inhaling anesthetics have been administered for this purpose.^2^^,^^30^ In a review of several surgeries, it was shown that administration of various hypotensive agents can be helpful in controlling the bleeding amount at the filed of oromaxillofacial surgery, endoscopy of sinuses, ear micro-surgery, backbone and nervous system surgeries, orthopedic surgeries, prostate surgery and also heart or liver transplants.^31^ However in the case of rhinoplasty, few studies have been conducted. So we decided to look into the effect of labetalol and nitroglycerin on the bleeding amount at operation field. 

Although the bleeding amount was slightly higher in the labetalol group and surgeon’s satisfaction with the operation field quality was slightly higher in the nitroglycerin group, but we did not find a significant difference between the two groups regarding these factors. The average rate of heart beats also did not show a significant difference between the two groups.

In agreement with our results, Eltringham *et al.* also did not find a significant difference between the effects of these two drugs on the bleeding amount in median ear surgery. They concluded that both agents are good candidates to induce hypotension and reduce the bleeding amount during this type of surgery.^25^ In a separate study, Goldberg and colleagues evaluated the effect of labetalol and nitroprusside (another NO-releasing drug like nitroglycerin) on hypotension induction in major surgeries.^23^ They showed that nitroprusside significantly increased the rate of heart beat but no difference was observed in heart output due to labetalol administration.^23^ In Yeasmeen *et al.*’s study, the effect of labetalol and nitroglycerin administration on the aforementioned factors was evaluated in elective backbone surgery in two groups of patients. In one group, 1000 µg of nitroglycerin was administered intravenously and the other group received 5 mg of labetalol. They found a significant difference in the rate of heart beat with a significantly higher score for surgery quality and lower bleeding amount in labetalol group.^32^ In another study on the hypotensive effects of labetalol and nitroprussid, Fahmy and colleagues found that nitroprusside significantly increased the heart rate and heart output (P<0.05). Although in both groups, the PO2 was reduced but unlike nitroprusside cases, labetalol induced hypotension which was continuous even after drug deprivation.^28^

Our study shows that there was no significant difference in qualitative factors of operation field and especially the bleeding amount, during rhinoplasty, and after the administration of labetalol compared to nitroglycerin administration. However the results of nitroglycerin use showed minor improvements in the case of operation field quality and bleeding amount over the nitroglycerin. Regarding these side effects, nitroglycerin may be a better choice to induce hypotension during rhinoplasty. However these data need confirmation with another study and a bigger sample size. 

## Note

Please cite this paper as:

Hadavi MR, Zarei Y, Tarogh S. Comparison of Effects of Labetalol and Nitroglycerine on Intraoperative Blood Loss and Surgical Field Quality in Rhinoplasty Surgery. World J Plast Surg 2015;4(1):60-65.
